# Evaluation of Ultrasound‑Guided Erector Spinae Plane Block Versus Oblique Subcostal Transversus Abdominis Plane Block in Laparoscopic Cholecystectomy: A Comparative Study

**DOI:** 10.5812/aapm-157680

**Published:** 2025-02-04

**Authors:** Moataz Salah Khalil, Michael Fayez Yousef Metias, Maged Salah Mohamed, Ahmed Abd Elmohsen Bedewy, Tarek I. Ismail

**Affiliations:** 1 Anesthesia, Intensive Care and Pain Management, Faculty of Medicine, Helwan University, Cairo, Egypt; 2 Anesthesia, Intensive Care and Pain Management, Faculty of Medicine, Cairo University, Cairo, Egypt

**Keywords:** Ultrasound, Erector Spinae Plane Block, Oblique Subcostal Transversus Abdominis Plane Block, Laparoscopic Cholecystectomy

## Abstract

**Background:**

Many inter-fascial plane blocks, including the oblique subcostal transversus abdominis plane (OSTAP) block and, more recently, the erector spinae plane (ESP) block, have been utilized as part of multimodal analgesia in numerous abdominal surgeries.

**Objectives:**

This study aimed to evaluate the impact of using the OSTAP block and the ESP block as components of a multimodal analgesic technique in individuals undergoing laparoscopic cholecystectomy (LC).

**Methods:**

This randomized, controlled, single-blinded clinical study was conducted on 50 individuals aged 20 to 60 years, of both genders, with American Society of Anesthesiology (ASA) grade I and II physical status, undergoing LC. Subjects were allocated using a computer-generated randomization table into two equal groups: Group A received an ultrasound (US)-guided ESP block, and group B received a US-guided OSTAP block.

**Results:**

The Visual Analog Scale (VAS), mean arterial pressure (MAP), heart rate (HR), and respiratory rate (RR) at 6, 8, and 10 hours were significantly higher in the OSTAP block group compared to the ESP block group (P < 0.05). The time to the first morphine dose was significantly longer in the ESP block group compared to the OSTAP block group (P = 0.001). The total amount of morphine used was significantly greater in the OSTAP block group compared to the ESP block group. The incidence of nausea and vomiting did not differ significantly between the groups.

**Conclusions:**

Bilateral US-guided ESP blocks provide superior and prolonged postoperative analgesia and require less morphine use compared to OSTAP blocks following LC.

## 1. Background

Laparoscopic cholecystectomy (LC) is a common surgical procedure that necessitates multimodal analgesia for enhanced pain management (1). Unmanaged postoperative pain has several implications, including patient dissatisfaction, progression to chronic pain, prolonged hospital stays, and increased healthcare costs (2). Current guidelines recommend a multimodal analgesia strategy, employing various pharmacological agents and localized analgesic approaches to achieve effective pain management and mitigate opioid-related adverse effects (3). Many inter-fascial plane blocks, such as the oblique subcostal transversus abdominis plane (OSTAP) block and, more recently, the erector spinae plane (ESP) block, have been utilized as components of multimodal analgesic techniques in several abdominal surgeries (4). Ultrasound (US) has facilitated the precise identification of fascial planes and the administration of local anesthetics (LA) for the safe execution of these blocks (5).

The OSTAP block is an effective regional anesthetic method for postoperative analgesia following LC. It is a variant of the transversus abdominis plane (TAP) blocks, with numerous randomized controlled trials examining its efficacy in LC (6). The ESP block is a newly identified peri-paravertebral plane block. Several randomized controlled trials have demonstrated that bilateral ESP blocks provide effective postoperative analgesia in LC (7). The mechanism of action of the ESP block remains unclear; however, it is understood that LA spreads to both the posterior and anterior aspects of the transverse process (TP), affecting the ventral and dorsal rami, resulting in sensory blockade over a wide area (8). Previous research has shown significant analgesic effectiveness of the ESP and OSTAP blocks in the context of elective LC, with few studies comparing them directly (9).

## 2. Objectives

This study aimed to evaluate the impact of using the OSTAP block and the ESP block as components of a multimodal analgesic technique in LC.

## 3. Methods

This randomized, controlled, single-blinded clinical study was conducted on 50 subjects aged 20 to 60 years, of both sexes, with American Society of Anesthesiology (ASA) grade I and II physical status, and a Body Mass Index (BMI) of ≥ 20 kg/m² and ≤ 35 kg/m², undergoing LC. The study was conducted following approval from the Ethics Committee of Helwan University Hospitals, Cairo, Egypt, and was registered with the trial ID: NCT06640062. Informed written consent was obtained from all patients. Exclusion criteria included known sensitivities or contraindications to the study medications, histories of psychological conditions and/or chronic pain syndromes, contraindications to regional anesthesia, severe respiratory, cardiac, hepatic, and renal disorders, and pregnancy.

### 3.1. Randomization and Blinding

The participants were randomly divided using a computer-generated randomization table into two equal groups: Group A received an US-guided ESP block, and group B received a US-guided OSTAP block. The study was single-blinded, as only the participants were blinded due to being under general anesthesia, while the researcher performing the block was aware of the group assignments. Each subject underwent a comprehensive history taking, physical examination, laboratory investigations [complete blood count (CBC), international normalized ratio, alanine transaminase, aspartate transaminase, urea, serum creatinine, and random blood sugar], and radiological investigations [electrocardiogram (ECG) and chest X-ray]. On the night before surgery, participants received instructions on how to describe pain using a Visual Analog Scale (VAS), where 0 represents no pain and 10 signifies the worst possible pain. Informed consent was obtained. Preoperative fasting was required for at least 6 hours for solid meals and at least 2 hours for water and clear fluids.

During general anesthesia in the operating room, monitoring included pulse oximetry (SpO_2_), non-invasive arterial blood pressure (NIBP), ECG, heart rate (HR), and capnography. Anesthesia was induced following preoxygenation with 100% oxygen (O_2_) using 2 mg/kg propofol and 1 μg/kg fentanyl. Endotracheal intubation was facilitated with 0.5 mg/kg atracurium, with additional doses of 0.1 mg/kg administered every 25 minutes. Each patient received intravenous (IV) dexamethasone 8 mg and ondansetron 4 mg to mitigate postoperative nausea and vomiting. Anesthesia was maintained using isoflurane in a 50% O_2_/air mixture, with an expired isoflurane concentration of 1.2, and ventilation settings were adjusted to maintain an end-tidal carbon dioxide (CO_2_) level of approximately 30 - 40 mmHg. Intravenous fentanyl was administered at a dose of 0.5 μg/kg if the HR or mean arterial pressure (MAP) of any patient exceeded a 20% increase from baseline values. Hemodynamic parameters, including HR, MAP, O_2_ saturation, and end-tidal CO_2_, were recorded prior to induction and every 15 minutes until the end of the operation. Upon completion of skin closure, isoflurane was discontinued, and reversal was achieved with an IV injection of 0.02 mg/kg atropine and 0.05 mg/kg neostigmine. After extubation, subjects were admitted to the post-anesthesia care unit (PACU).

### 3.2. Performing the Ultrasound-Guided Block

The two blocks were conducted under full aseptic precautions following the induction of anesthesia and 15 minutes prior to the skin incision. The blocks were performed using a Mindray Diagnostic Ultrasound System Model Z60 (Mindray Bio-Medical Electronics, Shenzhen, China) portable US machine and a high-frequency linear probe (6 - 13 MHz). A 21-gauge, 10 cm long nerve-blocking needle (Stimuplex, B-Braun Melsungen, Germany) was used to perform the regional block. The LA mixture consisted of 20 mL of 0.5% bupivacaine, 10 mL of 2% lidocaine, and 10 mL of normal saline, resulting in a total volume of 40 mL. Twenty milliliters of this mixture was administered to each side.

### 3.3. Oblique Subcostal Transversus Abdominis Plane Block Technique

**Figure 1.**
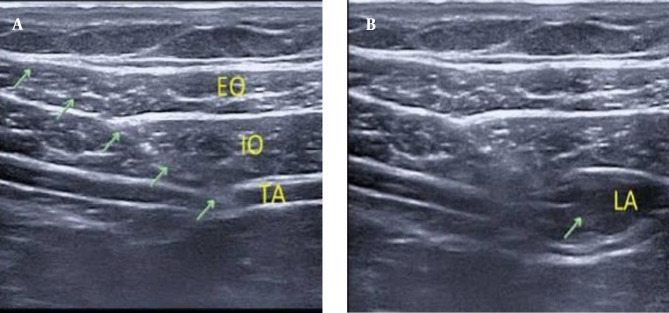
Oblique subcostal transversus abdominis plane (OSTAP) block A, with needle in place (green arrows); and B, after injection of local anesthetics. EO, external oblique muscle; TA, transversus abdominis muscle; IO, internal oblique muscle; LA, local anesthetics.

With the patient in the supine position, the linear probe is placed transversely just below the costal margin to identify the rectus abdominis, transversus abdominis, and both external and internal oblique (EO and IO) muscles from medial to lateral along the costal margin. At the lateral border of the rectus abdominis, the needle is inserted in-plane from medial to lateral, with the subsequent administration of 20 mL of the LA mixture into the fascial plane between the IO and transversus abdominis muscles along the oblique subcostal line. The block was replicated on the opposite side with the same volume (Figure 1). 

### 3.4. Erector Spinae Plane Block Technique

**Figure 2.**
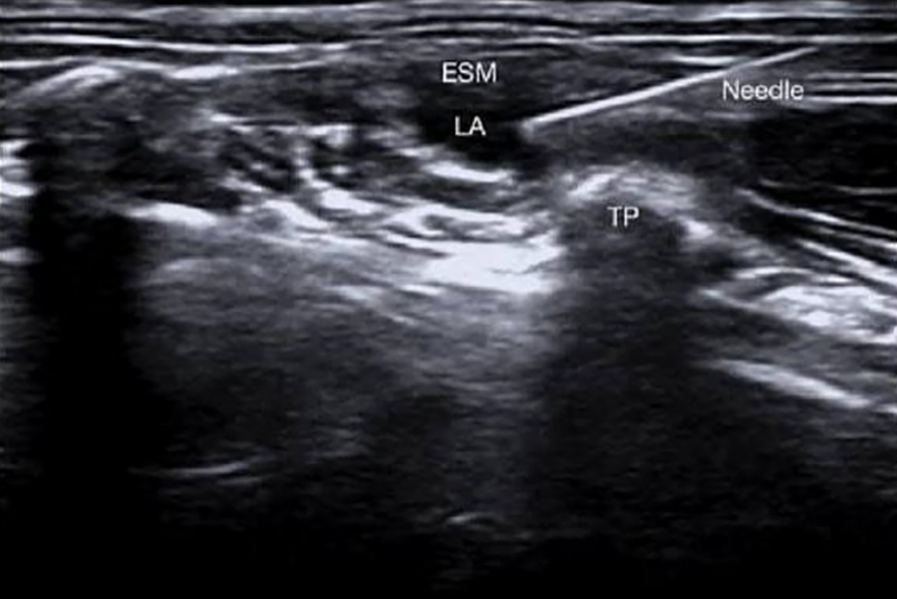
Erector spinae block. ESM, erector spinae muscle; TP, transverse process; LA, local anesthetics.

The blocks were conducted at the level of the T7 spinous process (SP) with the patient in the lateral position and the arm abducted. Using US, the T7 TP is identified by counting from the 12th rib. The US probe is positioned 2 - 3 cm laterally to the SP of T7 and situated over the TP of T7/T8 in the parasagittal longitudinal plane, with the erector spinae muscle (ESM) visualized over the TP. The needle is then inserted and advanced in-plane from cephalad to caudad until the needle tip contacts the TP of T7. Following hydrodissection with 2 mL of isotonic saline, which elevates the ESM, 20 mL of the LA mixture is administered after several negative aspirations. The same procedure was performed on the opposite side (Figure 2). 

After extubation and transfer to the PACU, standard analgesia, consisting of ketorolac 30 mg and acetaminophen 1 g, was administered to all patients via intravenous infusion every 8 hours for the first 24 hours postoperatively. Postoperative pain was assessed using the VAS at 30 minutes and at 2, 4, 6, 8, 12, 16, 20, and 24 hours. If patients reported pain with a VAS score ≥ 3, rescue analgesia in the form of morphine boluses was administered at a dose of 0.05 mg/kg as needed. The time until rescue analgesia and the total dose of morphine administered to each patient during the first 24 hours postoperatively were recorded.

The main outcomes were pain intensity measured by the VAS score at 30 minutes and at 2, 4, 6, 8, 12, 16, 20, and 24 hours after surgery, as well as the duration until the administration of rescue analgesia. The secondary outcomes included the total amount of morphine administered to each participant during the first 24 hours after surgery, in addition to morphine-related adverse effects such as the frequency of vomiting and nausea, respiratory depression [respiratory rate (RR) < 10, decreased arterial oxygen saturation < 90%, or increased arterial carbon dioxide > 50], pruritus, bradycardia (HR < 60 bpm), and urine retention.

Adverse effects of LA were also monitored, including circumoral numbness, lightheadedness, tongue paresthesia, sleepiness, irritability, muscular twitching, convulsions, hypotension (decrease in blood pressure > 20% of baseline), bradycardia, cardiac arrest, and any indications of complications from the block procedures (e.g., local site infections, hematoma formation, bowel perforations, and pneumothorax).

Complications identified by the researcher or reported by patients were treated accordingly. For example, nausea and vomiting were treated with intravenous ondansetron 4 mg once daily; respiratory depression was managed with supportive oxygen therapy up to mechanical ventilation when needed; bradycardia was treated with 0.01 mg/kg intravenous atropine; and hypotension was managed with supportive intravenous crystalloid infusion and intravenous ephedrine 5 mg bolus if required.

### 3.5. Sample Size Calculation

Using OpenEpi with a power of test at 80% and a confidence interval of 95%, the total sample size is 50 patients (25 in each group). The mean VAS score in the initial 24 hours after surgery in the ESP block group is 0.58, while in the OSTAP block group, it is 1.7 (10).

### 3.6. Statistical Analysis

Statistical analysis was conducted using SPSS version 26 (IBM Inc., Chicago, IL, USA). The Shapiro-Wilk test and histograms were used to evaluate the normality of the data distribution. Quantitative factors were represented as mean and standard deviation (SD) and compared across the two groups using the unpaired Student's *t*-test. Qualitative factors were represented as frequencies and percentages (%) and analyzed using the chi-square or Fisher's exact test, as appropriate. A two-tailed P-value < 0.05 was considered statistically significant.

## 4. Results

**Figure 3.**
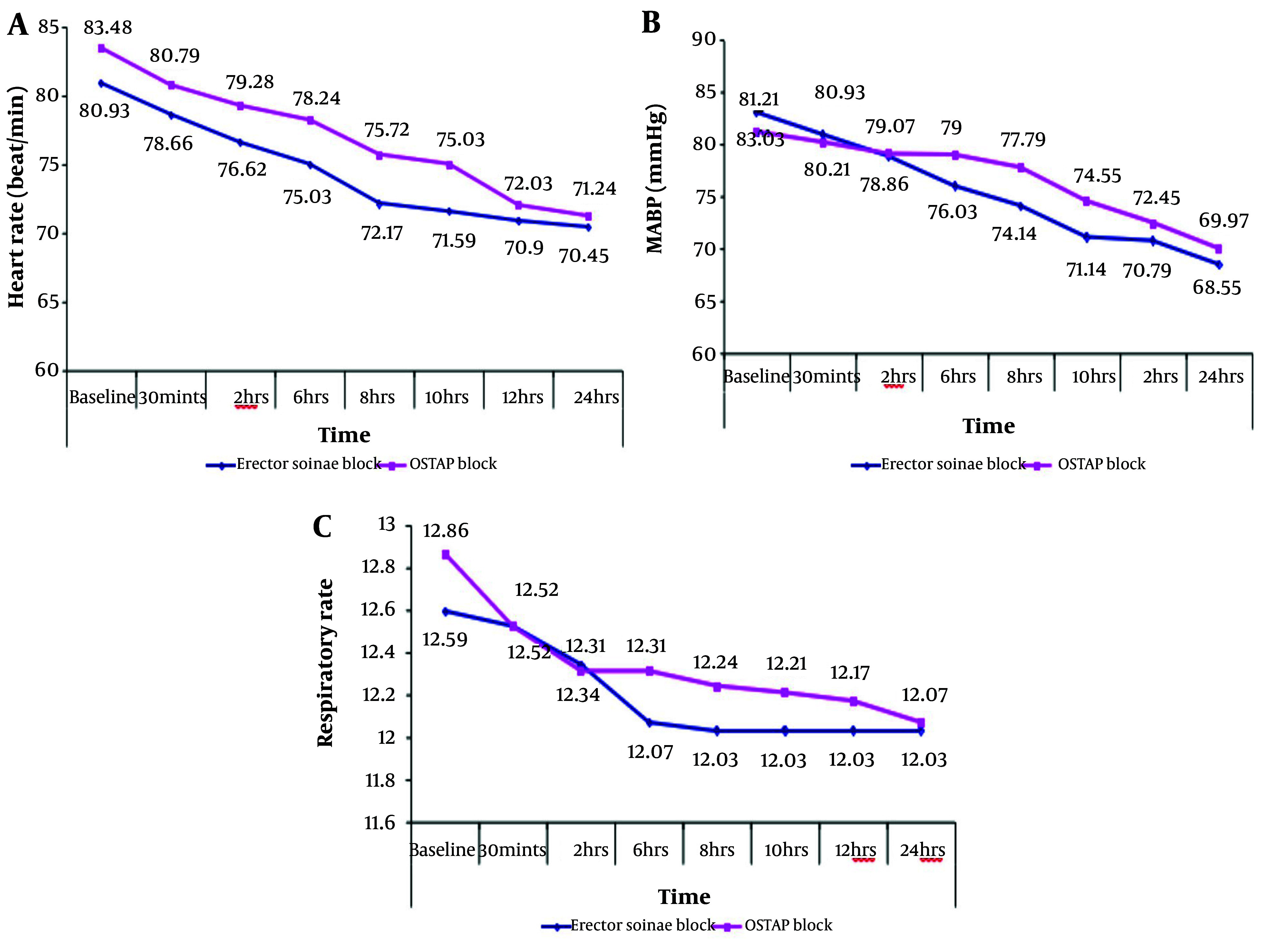
A, HR; B, mean arterial blood pressure; and C, RR between studied groups. HR, heart rate; RR, respiratory rate.

Demographic data did not significantly vary between the two groups (Table 1). The MAP, HR, and RR at 6, 8, and 10 hours were significantly higher in the OSTAP block group compared to the ESP block group (P < 0.05). The MAP, HR, and RR at baseline, 30 minutes, 2 hours, 12 hours, and 24 hours did not significantly differ between the two groups (Figure 3). The VAS scores at 6, 8, and 10 hours were significantly higher in the OSTAP block group compared to the ESP block group (P < 0.05). The VAS scores at 30 minutes, 2 hours, 4 hours, 12 hours, 16 hours, 20 hours, and 24 hours did not significantly differ between the groups (Table 2). The time to the first morphine dose was significantly longer in the ESP block group compared to the OSTAP block group (P = 0.001). The total amount of morphine was significantly higher in the OSTAP block group compared to the ESP block group (Table 3). The incidence of vomiting and nausea did not significantly differ between the two groups (Table 4). 

**Table 1.** Comparison Between Erector Spinae Plane Block and Oblique Subcostal Transversus Abdominis Plane Block Regarding Demographic Data ^a^

Variables	ESP Block (n = 25)	OSTAP Block (n = 25)	P-Value
**Age (y)**	39.14 ± 11.13	38.07 ± 12.33	0.730
**Gender**			1.000
Male	14 (48.3)	14 (48.3)	
Female	15 (51.7)	15 (51.7)	
**Weight (kg)**	73.52 ± 7.91	69.07 ± 10.29	0.070
**Height (m)**	171.76 ± 6.46	168.79 ± 7.87	0.122
**BMI (kg/m** ^**2**^ **)**	25.08 ± 1.73	24.95 ± 1.71	0.773
**ASA**			0.570
I	19 (65.5)	21 (72.4)	
II	10 (34.5)	8 (27.6)	
**Total time of surgery (min)**	132.93 ± 9.37	131.62 ± 9.93	0.607

Abbreviations: ESP, erector spinae plane; OSTAP, oblique subcostal transversus abdominis plane; BMI, Body Mass Index; ASA, American society of anesthesiologists.

^a^ Values are expressed as mean ± SD or No. (%).

**Table 2.** Comparison Between Erector Spinae Plane Block and Oblique Subcostal Transversus Abdominis Plane Block Regarding Visual Analogue Scale ^a^

Variables	ESP Block (n = 25)	OSTAP Block (n = 25)	P-Value
**30 (min)**	1.34 ± 0.48	1.45 ± 0.51	0.430
**2 (h)**	1.24 ± 0.44	1.48 ± 0.51	0.057
**4 (h)**	1.34 ± 0.48	1.52 ± 0.51	0.191
**6 (h)**	1.38 ± 0.49	1.83 ± 0.60	0.003 ^b^
**8 (h)**	1.52 ± 0.51	2.38 ± 1.27	0.001 ^b^
**10 (h)**	1.41 ± 0.50	2.21 ± 1.35	0.004 ^b^
**12 (h)**	1.48 ± 0.51	1.62 ± 0.68	0.384
**16 (h)**	1.69 ± 0.54	1.69 ± 0.47	1.000
**20 (h)**	1.72 ± 0.53	1.72 ± 0.45	1.000
**24 (h)**	1.66 ± 0.48	1.86 ± 0.35	0.068

Abbreviations: ESP, erector spinae plane; OSTAP, oblique subcostal transversus abdominis plane.

^a^ Values are expressed as mean ± SD.

^b^ Significant P-value < 0.05.

**Table 3.** Comparison Between Erector Spinae Plane and Oblique Subcostal Transversus Abdominis Plane Block Regarding Time and Total Amount of Morphine ^a^

Variables	ESP Block (n = 25)	OSTAP Block (n = 25)	P-Value
**Time of first morphine dose (h)**	15.25 ± 1.50	9.86 ± 2.01	0.001 ^b^
**Total amount of morphine (mg)**	4.45 ± 0.42	5.44 ± 0.37	0.003 ^b^

Abbreviations: ESP, erector spinae plane; OSTAP, oblique subcostal transversus abdominis plane.

^a^ Values are expressed as mean ± SD.

^b^ Significant P-value < 0.05.

**Table 4.** Comparison Between Erector Spinae Plane and Oblique Subcostal Transversus Abdominis Plane Regarding Nausea and Vomiting ^a^

Variables	ESP Block (n = 25)	OSTAP Block (n = 25)	P-Value
**Nausea**	2 (6.9)	6 (20.7)	0.128
**Vomiting**	1 (3.4)	4 (13.8)	0.160

Abbreviations: ESP, erector spinae plane, OSTAP, oblique subcostal transversus abdominis plane.

^a^ Values are expressed as No. (%).

## 5. Discussion

Managing postoperative pain after LC is a challenging task. This study aimed to evaluate the impact of using the OSTAP block and the ESP block as components of a multimodal analgesic technique among individuals undergoing LC. Our findings indicated that bilateral US-guided ESP block offers enhanced and prolonged postoperative analgesia while reducing morphine use compared to the OSTAP block following LC. The utilization of regional nerve blocks has increased, yielding positive outcomes by reducing the need for additional analgesics (11), with a subsequent reduction in drug-related adverse effects (12). The US-guided OSTAP block is a straightforward method that reduces postoperative pain and opioid use; however, it does not provide relief from visceral pain (13). The US-guided ESP block is regarded as an alternative technique that delivers effective postoperative analgesia for breast and thoracic surgeries at the T4-5 level and abdominal surgeries at the T7-10 level. The ESP block alleviates visceral and somatic discomfort by impacting the ventral ramus and rami communicantes, which comprise sympathetic nerve fibers, as the LA disseminates via the paravertebral region (14). Our findings indicate that the US-guided bilateral single-shot ESP block markedly reduced VAS ratings at postoperative intervals and was substantially superior at 6, 8, and 10 hours in contrast to the OSTAP block.

The diminished efficacy of the OSTAP block may be attributed to anatomical abnormalities that hinder the diffusion of LAs, in addition to the inconsistent segmental origins of anterior abdominal wall nerves, which restrict the application of the OSTAP block in upper abdominal interventions. Additionally, OSTAP blocks have demonstrated efficacy in alleviating parietal pain but not visceral pain (15). Hamed et al. (16) discovered that the VAS score postoperatively was markedly elevated in the control group during the first 12 hours post-surgery and similar to the ESP block following total abdominal hysterectomy. Elsayed Goda and Eldahshan (17) determined that VAS ratings were substantially reduced in the US-guided PVB group at immediate postoperative, 2, 6, and 24 hours compared to the TAP block after total abdominal hysterectomy. Limited studies have assessed the timing for the first demand of morphine during ESP and TAP blocks. The present research indicates that the time to the need for the first morphine administration was 15.25 ± 1.5 minutes in the ESP group and 9.86 ± 2.01 minutes in the OSTAP group, demonstrating a substantial increase in the ESP group. The results from a case series conducted by Luis-Navarro et al. (18) indicated that the initial rescue analgesic was necessary only at 16 hours post-ESP block. Hamed et al. (16) observed that bilateral ESP block significantly reduced fentanyl usage 24 hours postoperatively in contrast to the control group following total abdominal hysterectomy. Furthermore, Gurkan et al. (19) indicated that the average morphine intake at 24 hours postoperatively was 5.6 ± 3.43 in the ESP group, 5.64 ± 4.15 in the OSTAP group, and 14.92 ± 7.44 in the control group. The findings validated our results, as the total morphine intake over 24 hours was 4.45 ± 0.42 in the ESP group, in contrast to 5.44 ± 0.37 in the OSTAP group.

Postoperative vomiting and nausea are common complications and represent an unfavorable response to opioids (20). Melnikov et al. (21) discovered that the incidence of vomiting and nausea was decreased in the PVB group compared to the TAP block group (4 participants required antiemetics in the PVB group versus 8 participants in the TAP block group). In our investigation, the frequency of vomiting and nausea was greater in the OSTAP group; however, this variation was not statistically significant when compared to the ESP group. No additional adverse effects from opioids or bupivacaine, nor any technique-related problems, were observed. The reasoning appeared as follows: Initially, the TAP block does not inhibit visceral pain fibers, and the location of the block has relatively poor vascularity, which reduces the likelihood of systemic side effects from LA (22). Secondly, the application of the US method reduces the incidence of complications associated with both blocks. The ESP block targets the musculofascial plane situated superficially to the TP, ensuring the needle tip is kept away from the pleura, major arteries, and distinct nerves. Ultimately, a small quantity of bupivacaine is used (23). Conversely, Mittal et al. (24) determined that the US-guided TAP block is a viable, minimally invasive method that may contribute to successful multimodal analgesia in morbidly obese individuals undergoing bariatric and abdominal procedures. Keller et al. (25) showed that novices achieve the requisite timing for executing a successful block with increasingly less instruction to securely and effectively position OSTAP blocks. The study's limitations included the absence of sensory assessment of patients, as both blocks were administered under general anesthesia; nevertheless, this did not impact the outcome. Consequently, we urge future investigations comparing the two blocks.

### 5.1. Conclusions

Bilateral US-guided ESP block offers enhanced and prolonged postoperative analgesia compared to the OSTAP block following LC. Additionally, the time to first rescue analgesia in the form of morphine was significantly longer in the ESP block group compared to the OSTAP block group. Furthermore, the total amount of morphine used for postoperative analgesia was substantially lower in the ESP block group compared to the OSTAP block group.

### 5.2. Recommendations

We recommend further investigations comparing the effects of the two blocks on sensory sensation before the induction of general anesthesia. This would aid in evaluating the impact of both blocks concerning intraoperative analgesia.

## Data Availability

Data is available upon reasonable request from corresponding author.

## Clinical Trial Registration Code:

NCT06640062.
